# Development of Dual-Cross-Linked AlgMA/HAMA Hybrid Hydrogels for Traumatic Wound Healing

**DOI:** 10.1021/acsomega.6c04561

**Published:** 2026-08-26

**Authors:** Simay Y. Rivas Rodriguez, Alime Sarıkaya, Esra Su, Cemal Özeroğlu

**Affiliations:** † Department of Chemistry, Faculty of Engineering, Istanbul University-Cerrahpasa, 34320 Istanbul, Turkey; ‡ Department of Chemistry, Faculty of Science, Eskisehir Technical University, 26555 Eskisehir, Turkey; § Department of Biotechnology, Institute of Health Science, Bezmialem Vakıf University, 34093 Istanbul, Turkey; ∥ Bezmialem Vakif University Experimental Application and Research Center Cell Culture Laboratory, 34093 Istanbul, Turkey; ⊥ Department of Aquatic Biotechnology and Genomics, Faculty of Aquatic Sciences, Istanbul University, 34134 Istanbul, Turkey

## Abstract

Traumatic injuries and uncontrolled, intense bleeding caused by surgery remain among today’s leading medical problems. Traumatic wounds are not only observed on the skin but also result from internal organ ruptures caused by explosions and firearms. At this point, severe hemorrhaging can lead to hypothermia, hemorrhagic shock, organ failure, and even death due to the loss of more than 40% of blood volume. Therefore, it is crucial to halt bleeding rapidly. In this study, an alginate derivative that supports platelet aggregation and a hyaluronic acid derivative that adheres to wet tissues and induces angiogenesis, thereby promoting vascularization, have been prepared. The derivatives of alginate and hyaluronic acid were subjected to free radical photopolymerization, allowing them to cross-link in the presence of visible light. The study aims to introduce a new biomaterial featuring sodium alginate and hyaluronic acid groups that demonstrates good mechanical strength, a high swelling capacity to stabilize bleeding in the environment, and effective hemostatic properties. The chemical characterization of biopolymers was analyzed using FTIR and NMR techniques. The mechanical properties, swelling behavior, and degradation profiles of visible light cross-linked hybrid hydrogels were systematically characterized. The biocompatibility of the produced hydrogels was also evaluated using MTT and scratch wound healing tests. In addition, hemolysis and blood coagulation tests were performed to investigate the hemocompatibility and hemostatic potential of visible light cross-linked hybrid hydrogels.

## Graphical abstract

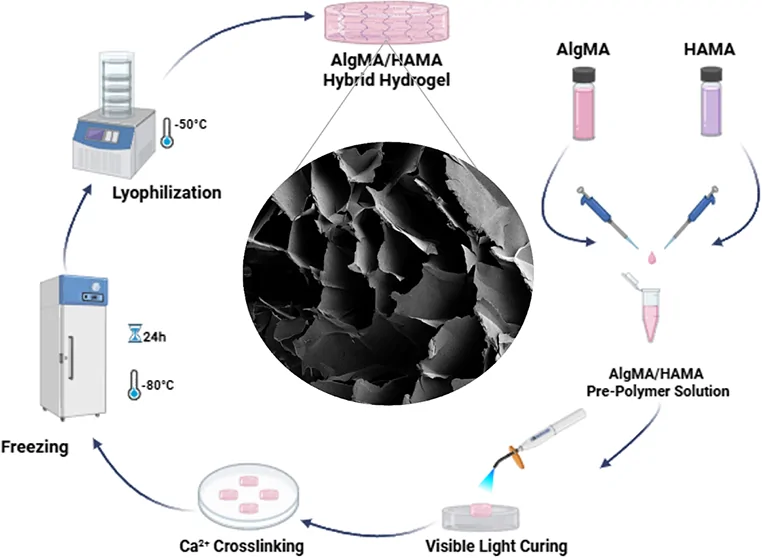

## 1 Introduction

Skin is the largest organ of the human body, accounting for approximately 10% of total body weight. Due to its direct contact with the external environment, it is highly susceptible to damage. Wounds are generally defined as disruptions in skin integrity and function arising from various causes such as trauma, surgical procedures, diabetes, and burns. The time required for wound healing can vary depending on the type of injury.^1,2^ Some wounds are characterized by their depth, excessive inflammatory response, and prolonged healing periods.^3^ Severe trauma, a leading cause of mortality worldwide, is responsible for the loss of more than 5.8 million lives each year. Approximately 40% of these fatalities result from uncontrolled hemorrhage, which is a major contributor to mortality in emergency and combat settings. Surgical procedures involving the spine, liver, cardiovascular system, and orthopedics can also give rise to significant bleeding. If not properly managed, increased blood loss can lead to numerous adverse outcomes,^4^ including hemorrhagic shock, hypothermia, hypotension, multiple organ failure, and acidosis.^5^ Once a diagnosis is made, key interventions include wound cleansing, dressing, infection prevention and control, pressure redistribution, restoration of tissue perfusion, and advanced cell-based therapies. These approaches may be applied individually or in combination to accelerate the healing process.^3^

One of the major challenges faced by modern medicine in the field of wound dressings is their lack of intrinsic properties that actively support wound healing. Conventional dressings currently in use often delay the healing of chronic and hard-to-treat wounds, contributing not only to economic burdens but also to numerous global health risks.^6^ The wound healing process, being inherently complex and dynamic, poses a significant challenge to healthcare systems worldwide, in addition to the associated social and psychological complications. Due to the inability of traditional dressings to promote wound healing, modern therapeutic wound dressings are being developed to create environments conducive to tissue regeneration.^7^ These advanced wound dressings are expected not only to support tissue regeneration but also to provide protection against bacterial infections arising from wound exudate or environmental sources. They must maintain a moist environment to prevent desiccation, possess a porous structure to allow oxygen diffusion without blocking air contact, and be biodegradable for easy application and removal. Consequently, natural biopolymers such as alginate, gelatin, chitosan, cellulose, silk fibroin, and hyaluronic acid are widely used in this field. Natural polymers like hyaluronic acid and alginate offer a moist environment that facilitates cell proliferation and migration, which in turn prevents scab formation and accelerates wound healing.

Photo-cross-linking is a simple approach to induce the formation of three-dimensional (3D) hydrogel networks. Photo-cross-linkable hydrogels demonstrate several advantages compared to other stimuli. For instance, photo-cross-linking is cost-effective, rapid, and simple way of fabricating 3D hydrogels with controlled shape, size, and spatial resolution. Photo-cross-linked cell-laden hydrogels have been successfully used for a number of applications, such as to study proliferation, endothelialization, and stem cell differentiation. A variety of cell-laden gels have been generated by methacrylate functionalization of different polymers such as alginate and HA and subsequent UV cross-linking of resultant polymer precursors.^8^

Hyaluronic acid, an endogenous polysaccharide, is a nonsulfated glycosaminoglycan. Its repeating disaccharide units bear numerous carboxyl and hydroxyl groups, which confer pronounced hydrophilicity. This property not only enhances cell adhesion but also promotes absorption of blood at the wound site, maintenance of a moist environment, prevention of tissue desiccation, and overall support of the healing process, owing to its high water-retention capacity.^9,10^ Furthermore, hyaluronic acid’s inherent swelling behavior facilitates cell attachment and migration; by promoting fibroblast proliferation, it stimulates collagen synthesis at the wound surface, making it one of the most suitable polysaccharides for wound healing applications.^11^ In addition to its physicochemical benefits, hyaluronic acid functions as a potent angiogenesis inducer.^12^ Angiogenesis, the sprouting of new capillaries from existing microvasculature—is central to both normal physiological processes (such as tissue and organ development and wound repair) and various pathological conditions. In injured tissues, hyaluronic acid triggers angiogenic signaling, thereby enhancing capillary formation within the wound bed. Both unmodified hyaluronic acid and its chemically derivatized forms are employed for these applications. Chemical modification of hyaluronic acid typically targets its hydroxyl and carboxyl groups,^13^ with the most common alteration being esterification of primary alcohol groups using methacrylic anhydride.^14^ Zhang et al. demonstrated that methacrylated hyaluronic acid (HAMA) induced angiogenesis in vitro within 3 days of culture.^15^ Consequently, photoinitiated free-radical polymerization of methacrylate-modified hyaluronic acid has become increasingly popular in tissue engineering over recent years.

Alginate, derived from brown algae, is a polyanionic polysaccharide composed of irregularly linked α-l-guluronic acid (G blocks) and β-d-mannuronic acid (M blocks) units via β-1,4-glycosidic bonds.^16^ The algal source, the relative proportion of G and M residues, and their sequential arrangement along the polymer chain all influence the physical properties of the resulting alginate.^9^ Owing to its capacity to promote adhesion of platelets and erythrocytes and to enhance wound-healing responses, alginate is widely employed as a supportive matrix for cell, enzyme, and peptide immobilization in tissue-repair applications, as well as in drug-delivery systems. Its intrinsic hydrophilicity further provides a moist environment that can mitigate wound formation and reduce bacterial infection. Although alginates represent a crucial class of polysaccharides for numerous biomedical applications, biopolymers generally undergo slow biodegradation in physiological settings.^17^ The alginate polymer can coordinate divalent cations (e.g., Ca^2+^, Mg^2+^) with carboxylate anions in its G-block regions, forming an “egg-box” structure. In Ca^2+^ cross-linked alginate hydrogels, interaction with blood triggers an ion-exchange process in which Na^+^ ions replace the bound Ca^2+^, releasing a high concentration of free Ca^2+^. These liberated calcium ions accelerate the activation of coagulation factors VII, IX, and X and promote platelet aggregation.^18^ As calcium functions as a vital cofactor in the coagulation cascade, its release from the hydrogel activates clotting pathways and thereby expedites the wound-healing process.^10,11^ Additionally, the physical cross-links provided by Ca^2+^ confer elasticity and mechanical resilience to the chemically cross-linked polymer network, enabling effective energy dissipation under mechanical stress.^19^ To enhance the stability of alginate hydrogels and prevent their dissolution, photo-cross-linkable hydrogels can be obtained by grafting methacrylate groups onto the carboxyl or hydroxyl side chains modifications that yield networks more stable than Ca^2+^ cross-linked alginate hydrogels. This esterification reaction can be carried out using glycidyl methacrylate, 2-aminoethyl methacrylate, or methacrylic anhydride. The methacrylated alginate (AlgMA) hydrogels thus produced can be cross-linked under UV–vis irradiation.^20^ Photopolymerization of the carbon–carbon double bond is frequently chosen to prepare hydrogels with low toxicity and adequate adhesive strength. Furthermore, alginate-based hydrogels synthesized by methacrylation of sodium alginate (SA) to yield AlgMA, followed by photoinitiator-mediated cross-linking, are widely employed in tissue engineering applications.^21^

Polymerization of these polymers using ultraviolet (UV) or visible light is a common strategy in the development of hydrogels for tissue engineering. Given the potential cytotoxic effects associated with UV exposure, photoinitiators that operate under visible light have been developed. Photo-cross-linkable polymers are typically acrylate derivatives.^22^ Methacryloyl groups can be activated for the photo-cross-linking of biopolymer solutions using a wide range of photoinitiators.^19^ For instance, Type I (cleavage-type) photoinitiators such as lithium phenyl-2,4,6-trimethylbenzoylphosphinate (LAP) are commonly employed for the formation of thiol-based hydrogels in the presence of live cells due to their favorable water solubility, cytocompatibility, and the ability of LAP to initiate rapid and efficient polymerization at low concentrations. Type I systems are sufficient on their own to initiate photopolymerization, while Type II photoinitiator systems (e.g., Eosin Y, Ruthenium complexes) require co-initiators or coagents to promote gelation kinetics.^23^

## 2 Materials and Methods

### 2.1 Materials

Methacrylic anhydride (MA), alginic acid sodium salt (SA) and the photoinitiator for photopolymerization, lithium phenyl-2,4,6-trimethylbenzoylphosphinate (LAP) were purchased from Sigma&Aldrich. Hyaluronic acid sodium salt (HA) was supplied by ACROSS. Dulbecco’s phosphate-buffered saline (DPBS) was purchased from BioWest. Ethanol (EtOH), sodium hydroxide (NaOH) and calcium chloride (CaCl_2_) of analytical grade were used without purification. The primary cell culture reagents, including Dulbecco’s Modified Eagle Medium (DMEM/F12), fetal bovine serum (FBS), and penicillin/streptomycin (P/S) antibiotics, were utilized. Phosphate-buffered saline (PBS), 0.25% Trypsin-EDTA, and trypan blue dye (0.4%) were obtained for the study. All sterile plasticware, including T-25 culture flasks and multiwell plates, were sourced from commercial suppliers. All chemicals used were of analytical grade.

#### 2.1.1 Synthesis of AlgMA and HAMA

Briefly, 2.5 g of SA was dispersed 100 mL deionized water under stirring until a homogeneous solution was obtained. After that, MA was added dropwise to the solution, and the pH of the solution was adjusted between 7.0 and 8.0 by adding 5 M NaOH. The reaction occurred out in the dark at room temperature for 72 h. After 72 h, the reaction solution was precipitated with 200 mL of EtOH. The precipitated substance was filtered and removed from the medium and dissolved again with 100 mL of deionized water. The solution obtained was dialyzed (MWCO 12–14 kDa) against deionized water to remove methacrylate salts and low molecular weight compounds formed in the synthesis reaction of AlgMA. Subsequently, it was kept at −80 °C and lyophilized for 48 h to obtain dry AlgMA.

HAMA was synthesized according to the literatüre.^24^ According to this literature, 1 g of HA was dispersed in deionized water under stirring overnight at room temperature. After 24 h, MA was added dropwise to the HA solution, and the pH of the solution was adjusted to 8.0 by adding 10 M NaOH solution. The methacrylated reaction of HA occurred out in the dark at 4 °C in an ice bath for 24 h. The obtained mixture was dialyzed (MWCO 12–14 kDa) against deionized water at 4 °C to remove methacrylate salts and low molecular weight compounds formed in the synthesis reaction of methacrylated HA. Subsequently, it was kept at −80 °C and lyophilized for 48 h to obtain dry AlgMA.

#### 2.1.2 Preparation of AlgMA and AlgMA-HAMA Hydrogels

Hybrid hydrogels are designed with a solid weight/solution volume ratio of 5% AlgMA/(1%/2%) HAMA. First, the PI–PBS solution was prepared for the 5% AlgMA solution. By using photoinitiator (PI) of lithium phenyl-2,4,6-trimethylbenzoylphosphinate (LAP) for photopolymerization, the prepared AlgMA solution, 0.1% (w/v) LAP (PI-system), was mixed at 50 °C for 1 h with the help of a heater-magnetic stirrer and magnet in hydrogel synthesis reaction of AlgMA. For the preparation of HAMA solution, two solutions were prepared in PBS medium with a solid weight/solution volume ratio of 1% and 2% HAMA (20 mg/mL PBS and 40 mg/mL PBS) in hydrogel synthesis reaction of AlgMA/HAMA. The solutions were mixed at 50 °C for 2 h in a heater-magnetic stirrer with the help of magnetic fish. Prepared solutions were mixed in equal volumes (1:1). A vortex was used for 2 min for the solution to mix. The prepared hydrogel solutions of AlgMA and AlgMA/HAMA was transferred into a glass tube with both ends open, after sealing one end with parafilm. The sample was then covalently cross-linked with visible light radiation (wavelength = 385–515 nm, intensity = 900–1300 mW/cm^2^, Valo Cordless Curing Light) for 240 s inside the glass tube. Subsequently, it was removed from the tube and immersed in 1 M CaCl_2_ solution for 5 min to allow for ionic cross-linking of the AlgMA biopolymer. After 5 min, cut into equal-sized pieces using a razor blade and the hydrogel taken from the solution was subjected to a gradual washing process and the residual chemicals released after the polymerization and excess ions from CaCl_2_ were separated. After the prepared hydrogel was frozen at −80 °C, the freeze-drying method was dried by lyophilization.

#### 2.1.3 Characterization of the AlgMA/HAMA Hydrogels

The structural analysis of AlgMA/HAMA hybrid hydrogels was performed using Fourier Transform Infrared (FTIR) spectroscopy. FTIR measurements were conducted with a PerkinElmer Spectrum FTIR 200-ATR model spectrophotometer. The cross-linked AlgMA/HAMA hydrogels were first frozen at −80 °C and subsequently lyophilized. The degree of cross-linking in the lyophilized AlgMA/HAMA hybrid hydrogels was determined based on the FTIR spectra obtained from the analysis.

To investigate the physical properties of AlgMA/HAMA hybrid hydrogels, the pregel solutions were transferred into the glass tube and immediately cross-linked. To obtain the dry cylindrical samples, cross-linked hydrogels were lyophilized. First, the dried samples were weighed and immersed in PBS solution at 37 °C for 24 h. Then swollen samples were weighed. The swelling ratio was calculated by the following equation

1 $$\mathrm{swelling}\;\mathrm{ratio}\;\left(\%\right)=\frac{\left(W_{t}-W_{0}\right)}{W_{0}}\times 100$$

Here *W*_0_ is the initial weight of dried hydrogel and *W*_t_ is the wet weight of the samples. The data displayed at the end of this test were the mean values of triplicate measurements.

The degradation behaviors of the AlgMA/HAMA hydrogels were assessed by immersing the completely dry and preweighed cylindrical samples in PBS solution at 37 °C for 6 w. At certain time intervals, the samples were extracted from the degradation medium and then lyophilized. The decrease in the sample weight was determined by considering the dry weight of samples before and after incubation. The degradation ratio was calculated by the following equation

2 $$\mathrm{degradation}\;\mathrm{ratio}\;\left(\%\right)=\frac{\left(W_{i}-W_{d}\right)}{W_{i}}\times 100$$

Here *W*_i_ is the initial completely dry weight of samples, and *W*_d_ is the dry weight after incubation at a particular data point. All samples were measured in triplicate.

The mechanical performance of the AlgMA/HAMA hydrogels was determined at room temperature by the Uniaxial compression test machine (Zwick Roell Z0.5 TH) equipped with a 500 N load cell. Prior to the test, initial compression contacts of 0.01 N were applied to ensure full contact between the gel and the plates. The analyses were carried out with cylindrical samples with a diameter of 8–10 mm and a height of 10 mm. For uniaxial compression measurements, hydrogel samples were compressed at various strain rates. The measurements were repeated 3 times to calculate the mechanical strength of the hydrogels. All measurements were carried out at a constant compression speed.

For uniaxial tensile tests, 20 N pneumatic grips and 6 bar pressure were used. The analyses were carried out by preparing 2 × 10 mm^2^ hydrogels in the form of plates. For uniaxial elongation measurements, the cross-linked-gel length and strain rate were set to 10 ± 1 mm and 1 min, respectively.

#### 2.1.4 Structural Analysis

The internal morphology and pore structure of the hydrogel scaffolds were examined by scanning electron microscope (SEM). AlgMA and HAMA polymers were characterized by ^1^H NMR analysis. Analysis was performed at 50 °C in D_2_O at 400 MHz.

#### 2.1.5 Cell Culture Studies

##### 2.1.5.1 MTT Analysis

The cytotoxicity of the AlgMA/HAMA hydrogels was evaluated via a direct contact assay using CCD 986Sk cells (1 × 10^5^ cells per well) in accordance with established protocols.^25^ Briefly, cells were seeded into 24-well plates and maintained under standard culture conditions until adherence. Following the removal of the initial culture medium, hydrogel samples were carefully placed in direct contact with the cell monolayer. The plates were then replenished with fresh medium and incubated at 37 °C in a 5% CO_2_ environment for 24 and 48 h. Gel-free wells served as the control group, and all experimental groups were studied in triplicate.

At the designated time points, cell viability was quantified using the MTT [3-(4,5-dimethylthiazolyl-2)-2,5-diphenyltetrazolium bromide] assay. After removing the hydrogels, the wells were treated with a fresh medium containing MTT reagent and incubated for an additional 3 h at 37 °C. This process facilitates the reduction of the yellow tetrazolium salt into purple formazan crystals by the mitochondrial activity of viable cells. Subsequently, the supernatant was replaced with 100 μL of dimethyl sulfoxide (DMSO) to solubilize the formazan precipitates. The metabolic activity, reflecting cell viability, was determined by measuring the optical density (OD) at 570 nm using a microplate reader. The percentage of cell viability was calculated according to the following equation

3 $$\mathrm{cell}\,\;\mathrm{viability}\;\left(\%\right)=\left(\frac{{\mathrm{OD}570}_{\mathrm{sample}}}{{\mathrm{OD}570}_{\mathrm{control}}}\right)\times 100$$

Here OD570_sample_ is the optical density of the experimental hydrogel group, and OD570_control_ is the optical density of the gel-free control group.

##### 2.1.5.2 In Vitro Wound Healing (Scratch) Assay Analysis

To evaluate the effects of AlgMA/HAMA hydrogels on cell migration, a scratch assay was performed using CCD-986Sk cells. The cells were seeded into 24-well plates at a density of (1.5 × 10^5^ cells per well) cells per well and cultured until they reached a confluent monolayer. A vertical linear scratch (wound) was created in the center of each well using a sterile 200 μL plastic pipet tip. To remove cellular debris, the wells were gently washed with phosphate-buffered saline (PBS).

Subsequently, the AlgMA/HAMA hydrogel samples were introduced to the scratched cell monolayers. The plates were then incubated under standard conditions (37 °C, 5% CO_2_) for 24 and 48 h. The migration of cells into the denuded area was monitored and captured using an inverted light microscope at 0, 24, and 48-h time intervals. The gap closure, representing the migration capacity, was quantified by measuring the distance and the wound area using ImageJ software. All experiments were performed in triplicate to ensure statistical reliability.

4 $$\mathrm{wound}\;\mathrm{healing}\;\left(\%\right)=\frac{\left(\mathrm{area}\times t_{0}-\mathrm{area}\times t_{x}\right)}{\mathrm{area}\times t_{0}}$$

Here the initial scratch wound area at 0 h and represents the remaining wound area measured at a designated time interval (*x* = 24 or 48 h).

#### 2.1.6 In Vitro Hemolysis Analysis

To evaluate hemocompatibility, erythrocytes were separated from fresh bovine whole blood by centrifugation at 3500 rpm for 5 min. Following plasma removal, the erythrocytes were washed three times with PBS and subsequently diluted in PBS at a 1:9 ratio. Disc-shaped lyophilized hydrogel samples were then mixed with the erythrocyte suspension at a 1:1 (v/v) ratio and incubated at 37 °C for 2 h. PBS and distilled water served as the negative and positive controls, respectively. After incubation, the mixtures were centrifuged at 3500 rpm for 10 min to pellet the intact erythrocytes. The resulting supernatants were collected, photographed, and transferred into a 96-well plate for further analysis. Subsequently, the absorbance (Abs) of the supernatant was recorded at 540 nm to detect the hemoglobin release using a microplate spectrophotometer and the hemolysis degree calculated. The percentage of hemolysis was determined using the following equation.^26^

5 $$\mathrm{hemolysis}\;\left(\%\right)=\frac{{\mathrm{OD}540}_{\mathrm{sample}}-{\mathrm{OD}540}_{\mathrm{negativecontrol}}}{{\mathrm{OD}540}_{\mathrm{positivecontrol}}-{\mathrm{OD}540}_{\mathrm{negativecontrol}}}\times 100$$

#### 2.1.7 In Vitro Blood Coagulation Analysis

To evaluate blood clotting time, citrated whole bovine blood was recalcified by mixing it with 0.1 M CaCl_2_ at a 9:1 (v/v) ratio, followed by vortexing for 5 s. Prior to the experiment, disc-shaped lyophilized hydrogel samples were placed at the bottom of the wells in a 96-well plate, while empty wells served as the control group. Immediately after recalcification, 50 μL of the activated blood mixture was added to each well. Clot formation was monitored by gently rinsing each well with 150 μL of PBS at 1 min intervals. The clotting end point was defined as the time point at which the aspirated PBS became clear, indicating the absence of free erythrocytes and the completion of blood coagulation. Subsequently, 100 μL of supernatant per sample was transferred to the 96-well plate, and the absorbans of samples was measured at 540 nm using a microplate spectrophotometer, and the blood coagulation index (BCI) values were calculated according to this equation^27^

6 $$\mathrm{BCI}\;\left(\%\right)=\frac{{\mathrm{Abs}}_{\mathrm{sample}}}{{\mathrm{Abs}}_{\mathrm{blankcontrol}}}\times 100$$

Ethical approval was not required for the in vitro hemocompatibility experiments because bovine blood was obtained from a commercial slaughterhouse, and no animals were used, handled, or sacrificed specifically for the purposes of this research.

#### 2.1.8 Statistical Analysis

All characterization tests were performed three times, and results are presented as mean ± standard deviation. Levene’s homogeneity test and Oneway ANOVA were performed using SPSS-31 (IBM SPSS Statistics) to determine whether the properties of AlgMA/HAMA hybrid hydrogel dressings showed a significant difference depending on the different ratios of HAMA. Statistical significance of the experimental data was considered to be at the *p* < 0.05 level.

In the characterization of AlgMA/HAMA hybrid hydrogel dressings, since the variance of all variables such as swelling degree, mechanical properties based on compression and tensile tests, cell viability and wound healing was found to be homogeneous according to the Levene’s test, multiple comparisons were performed using Post Hoc Tukey HSD tests.

## 3 Results and Discussion

The hybrid hydrogels are composed of two widely used biopolymers, alginate and hyaluronic acid, both modified with vinyl groups. The methacryloyl groups can be activated using a broad range of photoinitiators for the photo-cross-linking of biopolymer solutions. The reaction mechanisms presented in Figure 1A.

**Figure 1:**
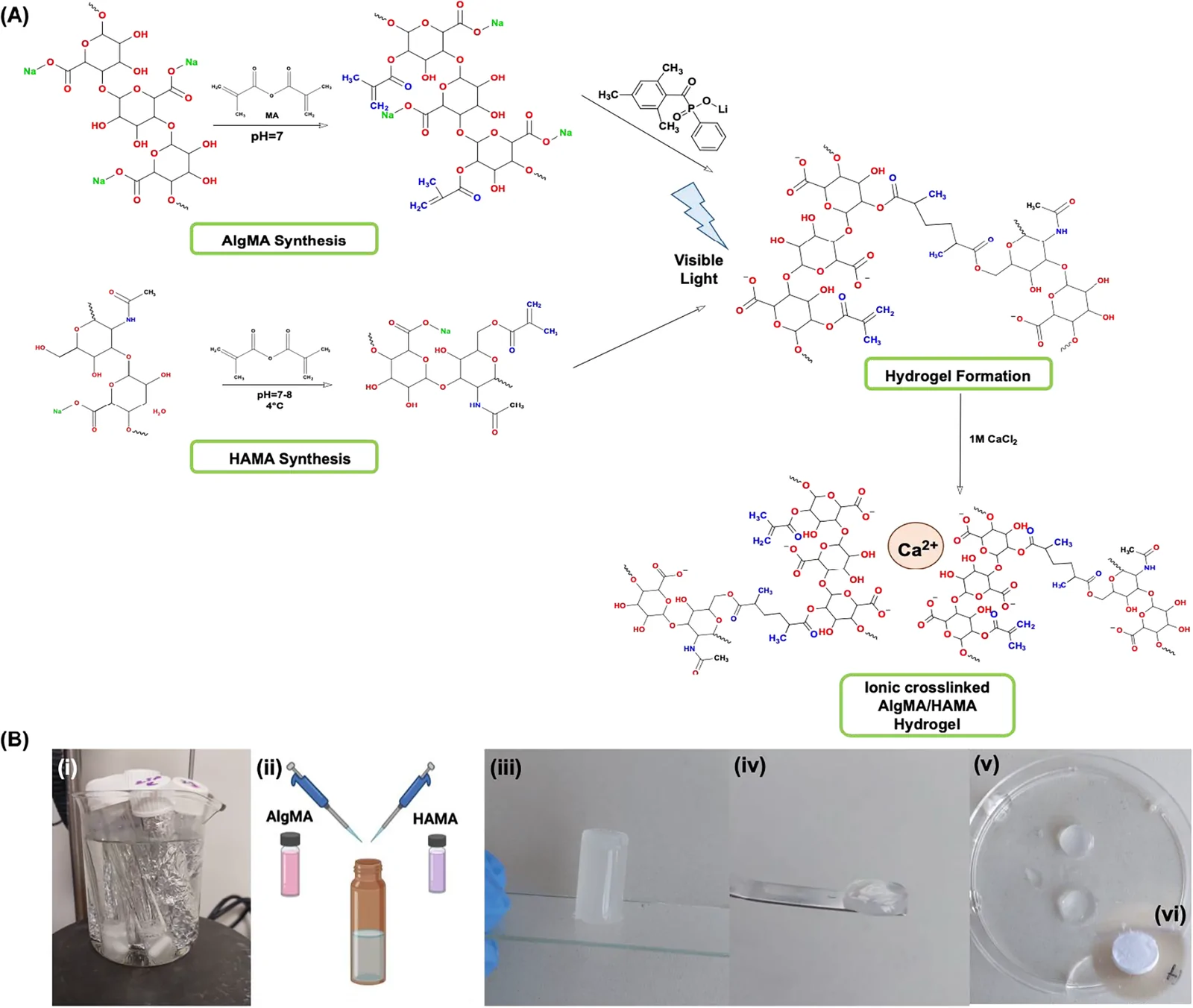
(A) Synthesis of AlgMA, HAMA and AlgMA/HAMA hybrid hydrogels mechanisms. (B) Macroscopic visualization of the AlgMA/HAMA hybrid hydrogel fabrication steps. (i) Preparation of the pregel precursor solutions under controlled heating and stirring. (ii) Schematic representation of blending the AlgMA and HAMA precursors. (iii) Free-standing cylindrical hydrogel demonstrating structural stability and shape retention immediately after visible-light photo-cross-linking. (iv) A photo-cross-linked hydrogel disc exhibiting mechanical integrity and ease of handling. (v) Macroscopic appearance of the swollen hydrogel discs, demonstrating their high fluid retention capacity and structural integrity without dissolving. (vi) Macroscopic appearance of the final lyophilized hybrid hydrogel matrix.

In this study, synthesized and characterized hybrid hydrogels composed of various HA with alginate. These hybrid hydrogels could potentially be used for applications for traumatic injuries healing. In the study, characterized the chemical and physical properties of the resulting hydrogels including swelling, compressive and tensile moduli, as well as biological properties such as cell viability and wound scratch assay.

HA is the primary natural extracellular matrix (ECM) component in various tissues. However, it can present several disadvantages when used as a single-component biomaterial. While HA is a primary ECM component, its nonadhesive nature limits its use in applications where cell spreading is required. Limitations of alginate hydrogels stem primarily from their mechanical weakness and rapid degradation behavior. To improve the physical and biological properties of AlgMA and HAMA, we fabricated AlgMA/HAMA hybrid hydrogels using different ratios of these two components.

### 3.1 Characterization of AlgMA and HAMA Polymers

To gain the photo-cross-linkable behavior to SA and HA biopolymers, they were functionalized with MA. Then, hydrogel dressings can be prepared with visible light. The obtained AlgMA and HAMA biopolymers were characterized by FTIR and ^1^H NMR analyses. The characteristic carbonyl units from the ester absorption peak at 1708 cm^–1^ was clearly revealed due to the presence of MA groups on the HAMA (Figure 2c). For further investigations, ^1^H NMR results of HAMA are presented in Figure 2d. According to the ^1^H NMR peaks, two specific peaks at 5.62 and 6.07 ppm were directed to double bond protons (C=CH_2_), and the band appeared at 1.87 ppm, described to methyl units neighboring to double bond (CH_3_–C=CH_2_), which do not exist in the HA.

**Figure 2:**
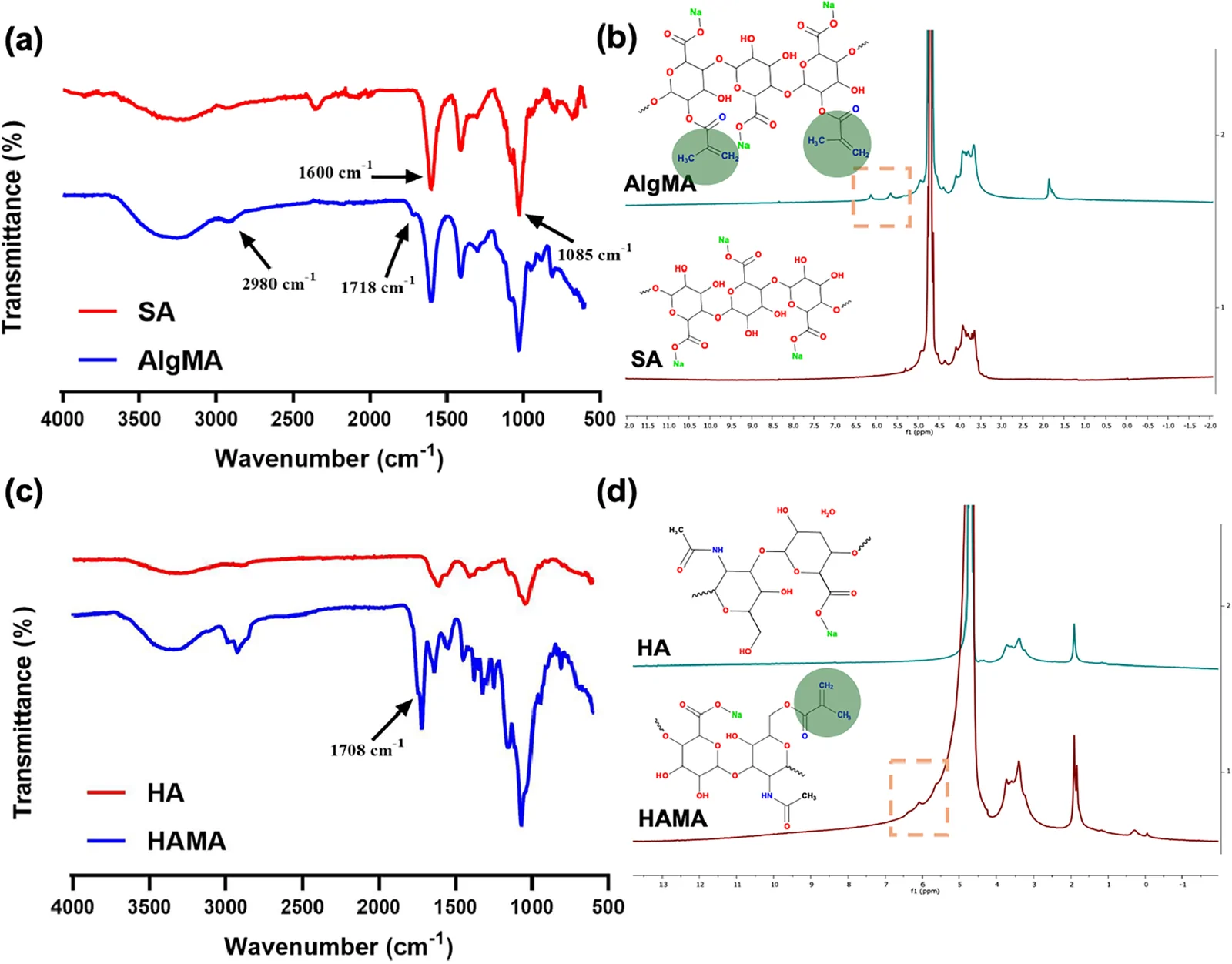
(a) FTIR-ATR and (b) ^1^H NMR results of sodium alginate and AlgMA. (c) FTIR-ATR and (d) ^1^H NMR results of sodium hyaluronate and HAMA.

For the characterization of AlgMA, FTIR and ^1^H NMR analyses were performed. In the FTIR spectra, AlgMA showed a peak at 1718 cm^–1^ attributed to ester absorption. According to the ^1^H NMR spectrum of AlgMA, two peaks have appeared at 5.66 and 6.12 ppm in contrast to spectrum (Figure 2b). These peaks can be assigned to ethylene group and characteristic of the methacrylate presence on the AlgMA backbone. In addition, at 1.85 ppm, methyl protons (−CH_3_) in the structure are seen. These results are evidence of the successful obtaining of functional polymers (Figure 2a).

### 3.2 FTIR Analysis of AlgMA/HAMA Hybrid Hydrogels

The successful cross-linking of AlgMA/HAMA hybrid hydrogel dressings was confirmed by FTIR analysis (Figure 3). In all spectra, the intensity of the band seen at ∼2900 cm^–1^ of the C=C bond in hybrid hydrogels diminished, demonstrating the success of obtaining cross-linked hydrogel structure.

**Figure 3:**
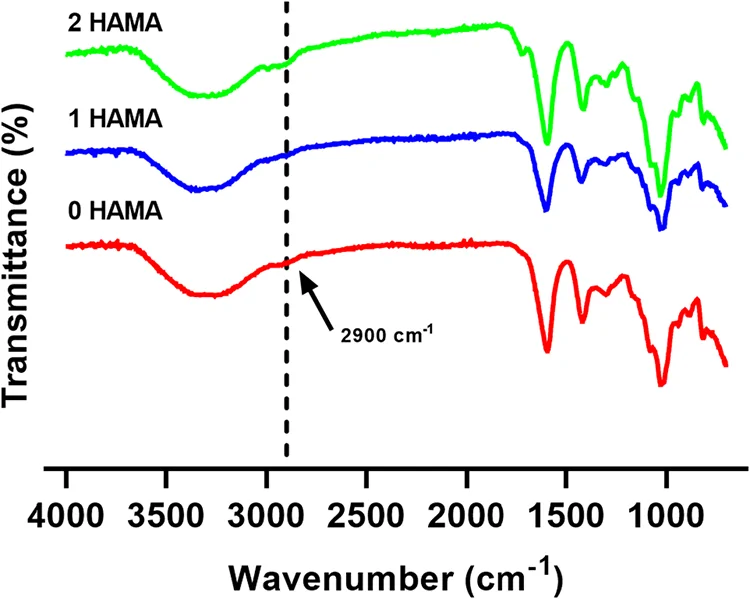
FTIR spectra of cross-linked AlgMA/HAMA hybrid hydrogels containing different ratio of HAMA.

### 3.3 Mechanical Properties of AlgMA/HAMA Hybrid Hydrogels

The mechanical properties of hydrogel wound dressings are extremely crucial to ensure the stability of the environment for cell proliferation. In addition, wound dressings intended for application on the wound surface should possess appropriate mechanical properties to accommodate human motion.^28^ The mechanical behaviors of hybrid hydrogels are influenced by cross-linking density, stiffness, pore size and porosity of the polymers. In this study, mechanical behaviors of hydrogels improved by increasing concentration of HAMA.

A compression test was carried out to determine the influence of the cross-linking due to HAMA amount on hybrid hydrogel dressings. The compressive stress versus strain plots of the AlgMA/HAMA hybrid hydrogel dressings recorded according to the dependence of the concentration are shown in Figure 4a.

**Figure 4:**
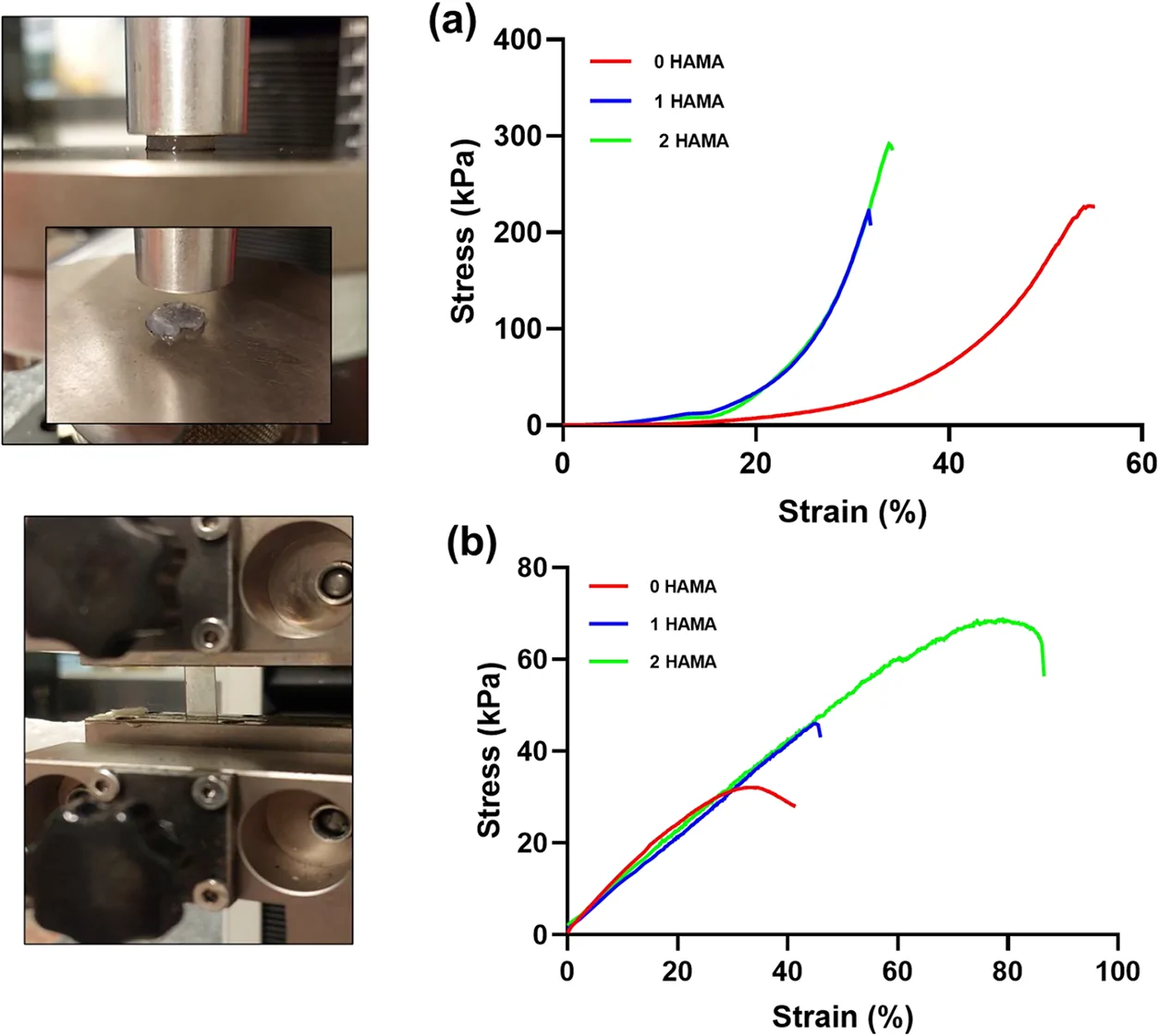
Compressive and tensile analysis results of cross-linked AlgMA/HAMA hybrid hydrogels. (a) Compression, (b) Tensile.

The material stiffness enhances as the polymer concentration increases, which results in an increase in the mechanical properties.^8^ At a 0 HAMA concentration, the pressure can vary from 20 to 60 kPa with the addition of 1 and 2 HAMA (Figure 4b). This is due to the increased cross-link density with HAMA concentration.

In compression tests, the Young’s modulus of hydrogel dressings E remained unchanged with the addition of 1 HAMA, while the modulus decreased significantly for the sample containing 2 HAMA. While the fracture stress, σ_f_ values did not change with HAMA content, the fracture strain, ε_f_ values showed a significant improvement with the addition of 2 HAMA. Although the Young’s modulus values obtained from tensile tests also appeared to decrease slightly with the addition of HAMA, this decrease was not significant at the 95% confidence interval. The fracture stress and strain values also decreased significantly with the addition of HAMA (Table S1).

Examining the stress–strain curves under compression, it is observed that the hybrid hydrogel becomes harder and more brittle with increasing HAMA concentration. Considering these values, a decrease in elongation from 50 to 60% to 30–40% is considered suitable for wound healing materials.^29^

**Figure 5:**
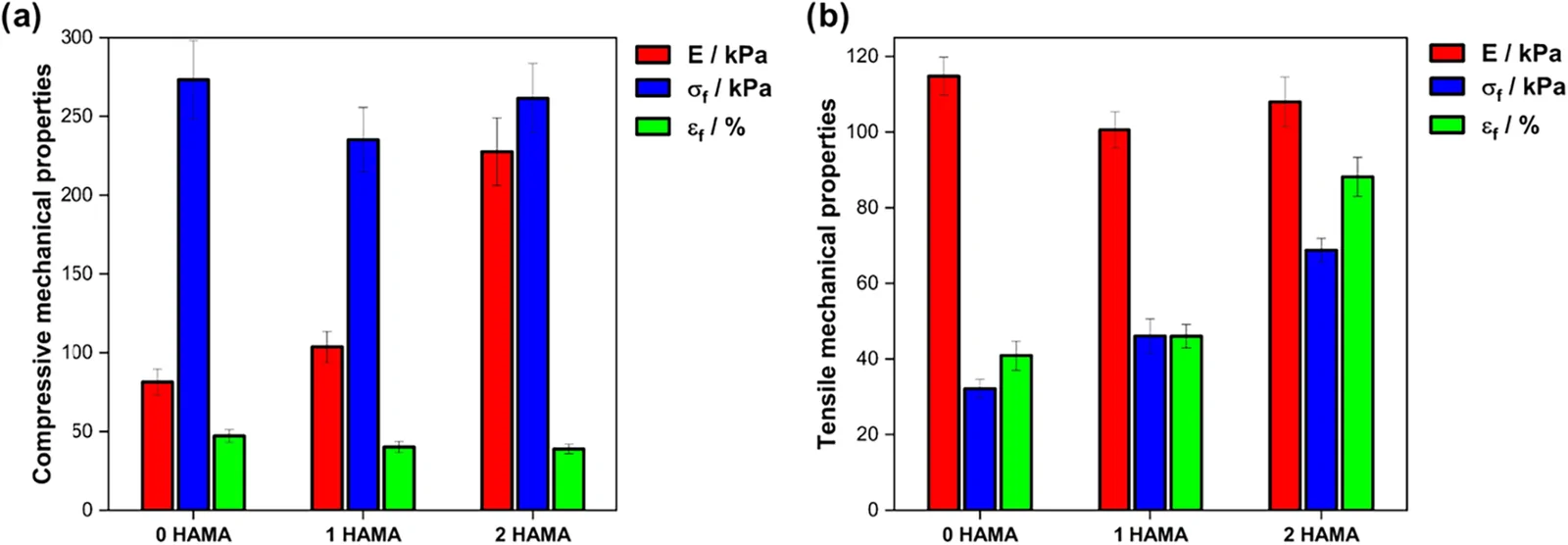
Mechanical properties obtained from compression (a) and tensile tests (b). Young’s modulus (E), fracture stress (σ_f_) and fracture strain (ε_f_) values are shown in red, blue and green bar graphs, respectively.

The mechanical performance of the hydrogels discussed above was determined in the prepared state. Similar exceptional mechanical properties are observed in their equilibrium swollen state at approximately 15% PBS, which can be calculated using the finite extensibility of the polymer chains.^30^ Schematic representations of the end-to-end distances of a network chain in the prepared state, at swelling equilibrium, and at the fracture point are given as *R*_0_, *R*_s_, and *R*_f_, respectively (Figure 6). Therefore, the deformation ratio λ_f_ at the fracture point of the postsynthesis hydrogels can be written as λ_f_ = *R*_f_/*R*_0_, while the chain expansion at the equilibrium swelling degree is given by α_s_ = *R*_s_/*R*_0_. Since α ∼ (swelling ratio)^1/3^ and λ_f_ = ε_f_ + 1, the λ_s,f_ values of the swollen hydrogels can be estimated using the swelling ratio and ε_f_ values of the hydrogels together with the formula as λ_s,f_ = λ_f_/α_s_. For the HAMA 0, HAMA 1 and HAMA 2 hydrogels, the fracture elongation ratios λ_s,f_ in the swollen state were calculated as 1.32 ± 0.02, 1.35 ± 0.03 and 1.71 ± 0.08 respectively, which indicates that they will fracture at more than 32% strain (Figure 5). Therefore, compared to the elasticity of the prepared hydrogels, their elasticity in the swollen state will not change significantly, as the stretching of the network chains with swelling remains almost unchanged.

**Figure 6:**
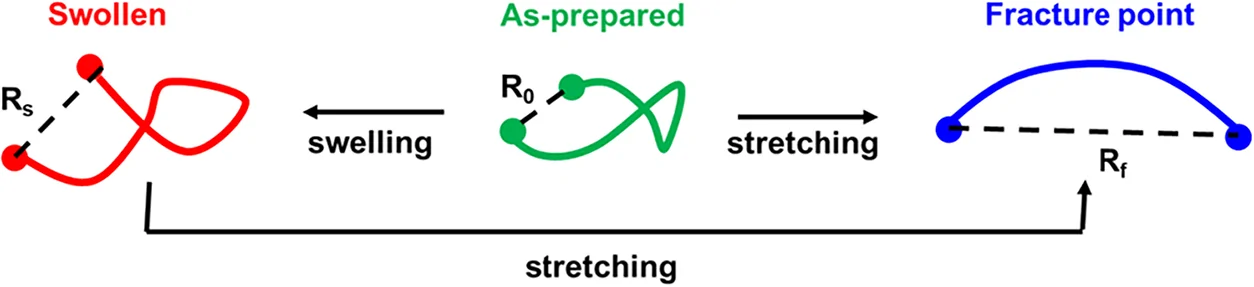
Scheme of a network chain during swelling and stretching. *R*_0_, *R*_s_, and *R*_f_ are end-to-end distances in the as-prepared state, swollen state, and at the fracture point, respectively.

### 3.4 Swelling and Degradation Behaviors of AlgMA/HAMA Hybrid Hydrogels

The degree of swelling is one of the important parameters for hydrogels designed as wound dressing. This is because the targeted wound dressing is expected to provide an appropriate level of moisture in the wound environment and help remove the exudate from the environment.^31^ In addition, cross-linked hydrogels are expected to decrease in the degree of swelling as the cross-link density increases. Figure 7a presents the swelling ratios of hybrid hydrogels in the PBS environment at 37 °C. When the results of the swelling capacity test are examined, it is seen that the cross-linked hydrogels reach the equilibrium swelling value at the 5 h. While faster and higher swelling behavior is observed in alginate hydrogels with less cross-link density, it is seen that the swelling kinetics slow down and reach balance more stably when the HAMA biopolymer, which increases the cross-link density, is added to the environment. This result supports the successful realization of photopolymerization.

**Figure 7:**
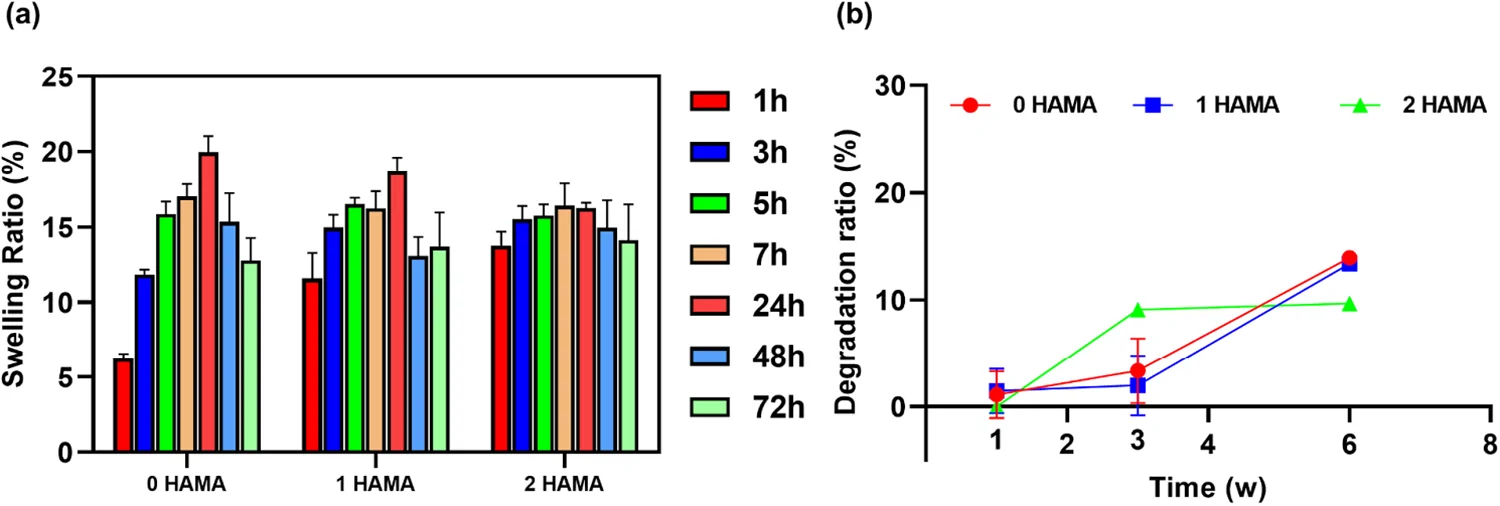
(a) Mass swelling ratio of AlgMA/HAMA hybrid hydrogels during 72h. (b) Mass degradation ratio of AlgMA/HAMA hybrid hydrogels for 1,3 and 6 weeks.

The degradation kinetics over a 6-week period in PBS at 37 °C are presented in Figure 7b. When examining the degradation rate, it is observed that the cross-linked hydrogels exhibit very low degradation capacity. It was determined that the degradation capacity decreased with increasing the density of the HAMA biopolymer, which increases the cross-link density in the medium. It is expected that wound dressings applied to traumatic injuries will continue to heal in the medium without exhibiting degradation.^32^

The surface morphology of the AlgMA/HAMA hybrid hydrogels, evaluated by SEM, is presented in Figure 8. Based on the surface morphology, the porous structure of the material enables the absorption of wound exudate from the wound bed while facilitating the transport of cellular metabolites and nutrients. This dual function contributes to an accelerated wound healing process.^33^ Cross-sectional SEM images further confirm the presence of porous architecture within the hybrid hydrogels.

**Figure 8:**
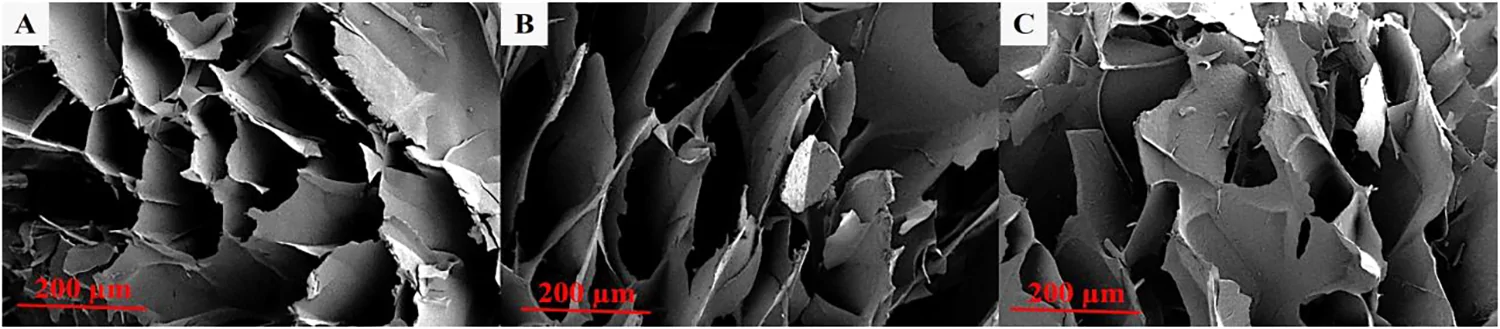
SEM cross section images of hybrid hydrogels with different ratios of HAMA. (A) 0 HAMA, (B) 1 HAMA, (C) 2 HAMA. Scale bar (200 μm) and magnification (150×) are indicated on the images.

### 3.5 Cell Culture

The long-term success of hemostatic agents, especially for internal applications, is strictly dependent on their cytocompatibility. The metabolic activity of cells in contact with the hydrogels was evaluated via MTT assay, and the results are presented in Figure 9c. All hydrogel formulations (0, 1, and 2 HAMA) exhibited cell viability significantly above the 80% threshold (indicated by the dashed line), which is the internationally recognized limit for noncytotoxicity according to ISO 10993–5. Statistical analysis revealed no significant decrease (*p* > 0.05) in viability as the HAMA concentration increased, confirming that the photopolymerization process and the presence of methacrylated groups do not induce toxic reactions. Furthermore, the statistically significant increase (*p* < 0.05) in viability from 24 to 48 h for all groups suggests that the hybrid hydrogels provide a conducive microenvironment that not only preserves cell health but actively supports cellular proliferation.

**Figure 9:**
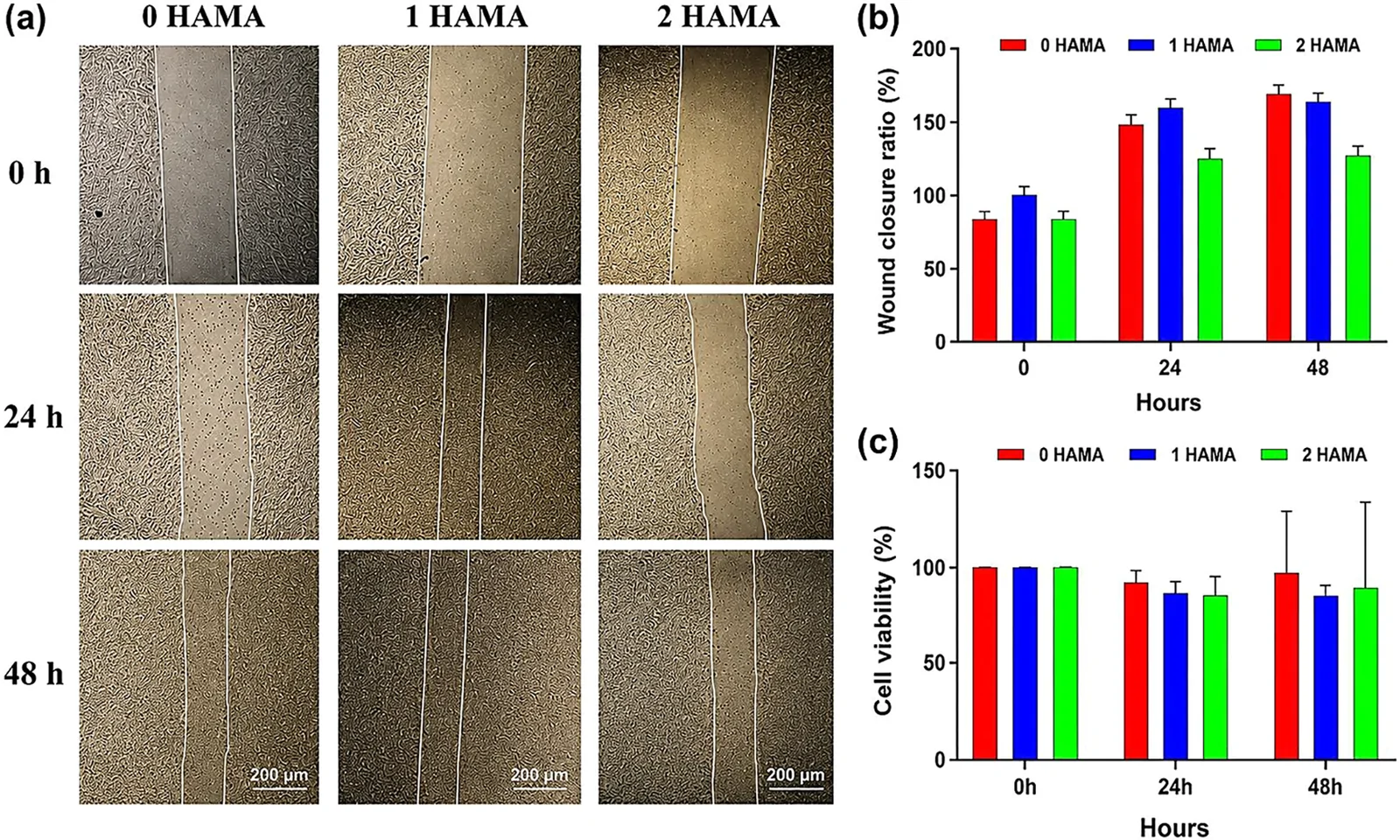
(a) Microscopic images taken at 0, 24, and 48 h of an in vitro wound scratch assay performed with CCD-986SK human skin fibroblast cells. (b) Wound closure rates of AlgMA/HAMA hybrid hydrogels in the wound scratch assay. (c) Cell viability rates of AlgMA/HAMA hybrid hydrogels after 24 and 48 h.

The regenerative potential of the hybrid scaffolds was qualitatively examined using an in vitro scratch wound assay. The optical micrographs in Figure 9a provide a visual representation of the cellular migration front at 0, 24, and 48 h. At the initial time point (0 h), a uniform and clear denuded area is observed across all samples. By 24 h, Figure 9a shows a progressive encroachment of cells into the gap, with the 1 HAMA group displaying a more advanced migration front compared to the control and 2 HAMA groups. By 48 h, the “wound” area in the HAMA-containing groups shows almost complete closure. This visual evidence confirms that the prepared biopolymers do not hinder cellular movement; instead, the hyaluronic acid-modified network likely facilitates chemotactic-like signals that encourage cells to repopulate the injured site.

The interaction between cells and hydrogels has significant influence on the progression of various cellular processes. Hydrogels, which are considered to have wound healing properties, are required to promote cell proliferation, differentiation and adhesion.^34^ Supporting cell migration plays an important role in wound regeneration. The fact that wound dressing plays a supportive role in cell migration is an effect that will shorten the healing process.^35^ Human fibroblast cells are very important both in terms of biocompatibility and their presence in many tissues in the human body.^36^ To validate the visual findings, the wound closure ratio was quantified and is shown in Figure 9b. A critical acceleration in healing was observed at 24 h, where the 1 HAMA group reached a statistically superior closure ratio (*p* < 0.01 vs 0 HAMA). This peak performance indicates that an optimum concentration of HAMA (1%) enhances cell motility, possibly through the activation of CD44-mediated signaling pathways inherent to hyaluronic acid. Interestingly, although the 2 HAMA group also showed significant closure (*p* < 0.05 vs control), its rate was slightly lower than 1 HAMA, which could be attributed to a higher cross-linking density providing a more rigid physical barrier. By 48 h, both the 0 HAMA and 1 HAMA groups achieved closure ratios exceeding 100%, indicating full gap coverage followed by continued proliferation. These quantitative results in Figure 9b, coupled with the biocompatibility data, demonstrate that the developed alginate-HA system is an effective dual-purpose biomaterial for rapid hemostasis and accelerated tissue repair.

While the current study successfully demonstrates the structural integrity and in vitro biocompatibility of the developed AlgMA/HAMA hybrid hydrogels, future in vivo validation will be essential to fully realize its clinical potential. In practical tissue engineering applications, static matrices often need to be upgraded into dynamic, multifunctional platforms to meet the complex demands of the native microenvironment. For instance, the therapeutic efficacy of our developed matrix could be significantly enhanced in future in vivo studies by integrating it with advanced, next-generation delivery systems. Recent pioneering studies have demonstrated that incorporating mRNA-loaded lipid nanoparticles (LNPs) into hydrogel or microneedle systems can efficiently orchestrate localized protein replacement, such as VEGF-A, to accelerate wound healing and angiogenesis without the instability issues of direct protein delivery.^37^ Furthermore, integrating stimuli-responsive elements-such as photothermally active nanomaterials that control the release of multiple biomimetic growth factors—has shown immense promise in guiding complex tissue regeneration and overcoming clinical challenges like infection.^38^ Therefore, utilizing our formulated scaffold as a base matrix to harbor such smart, nucleic-acid–based therapeutic delivery systems represents a highly promising future trajectory for its practical application in regenerative medicine.

### 3.6 In Vitro Hemolysis Analysis

Evaluating the blood contact safety and membrane disruption potential of any newly developed material is a crucial prerequisite for acute hemostatic and internal wound healing applications. For this purpose, an in vitro hemolysis experiment was performed using fresh bovine whole blood, and the results are shown in Figure 10. Visually, the supernatant fluids obtained after centrifugation of erythrocytes incubated with hybrid hydrogels (0, 1, and 2 HAMA) exhibited a clear, extremely light pink transparency. This macroscopic appearance was quite similar to the negative control (PBS) and contrasted sharply with the dark red, homogeneous lysis observed in the positive control (distilled water). Quantitatively, the pure alginate hydrogel matrix (0 HAMA) exhibited mild hemolytic activity (9.23%), showing a statistically significant difference compared to the negative control (*p* < 0.01). This mild membrane-destabilizing effect of pure alginate formulations on erythrocytes has been previously documented in the literature and is generally associated with tight ionic interactions at the cell-biopolymer interface.^39^ Remarkably, covalent hybridization of the alginate backbone with methacrylate hyaluronic acid (HAMA) derivatives provided severe, dose-dependent protection against erythrocyte rupture. Integration of 1% HAMA significantly reduced the hemolytic effect, bringing the rate down to 4.01% (*p < 0.05, compared with 0% HAMA). According to international standards set by ISO 10993–4, any biomaterial producing a hemolysis rate below the critical threshold of 5% is classified as nonhemolytic and safe for medical interventions (**ISO 10993–4:2017)**.^40^ More importantly, increasing the HAMA content up to 2% optimized the compatibility profile to a higher level; Here, the 2 HAMA hydrogel formulation recorded a hemolysis rate of only 1.19%, showing extremely high statistical significance (***p < 0.001) compared to the pure 0 HAMA control. Furthermore, statistical analysis confirmed that both the 1 HAMA and 2 HAMA formulations showed no significant difference (ns, *p* > 0.05) when directly evaluated with the negative control (PBS). This statistical equivalence (ns) highlights that the visual and chemical properties of the cross-linked network protect red blood cells from osmotic or physical stress and successfully eliminate the underlying cytotoxicity of crude biopolymers.^41^ The exceptional heme compatibility demonstrated by the 1 HAMA and 2 HAMA networks confirms that these hybrid matrices can function as highly safe, biosafe surgical sealants that can closely interact with bleeding tissue without causing systemic hematological toxicity or intravascular hemolysis.

**Figure 10:**
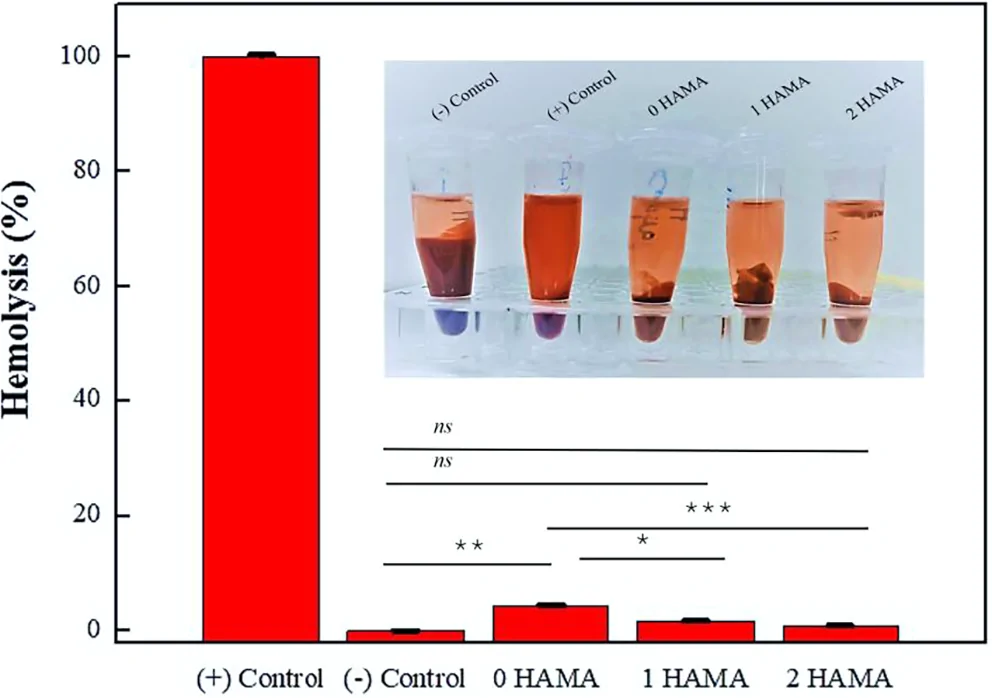
In vitro hemolysis assay of the hybrid hydrogels. Inset shows the visual macrographs of the centrifuged tubes. Data presented as mean ± SD (*p < 0.05,**p < 0.01, ***p < 0.001, and *ns*: not significant).

### 3.7 In Vitro Hemostatic Performance (Blood Clotting Index-BCI)

To evaluate the rapid coagulation efficiency of the synthesized gels containing 0 HAMA, 1 HAMA, and 2 HAMA, and to understand the hemostatic mechanism, a quantitative BCI test was performed using fresh bovine whole blood. In this test, a lower BCI percentage is directly associated with superior and faster blood coagulation kinetics, because a stable clot physically traps erythrocytes and prevents their lysis into the surrounding aqueous medium upon addition of water. As shown in Figure 11, the macroscopic presentation of the centrifuged supernatants provides clear experimental evidence of the hemostatic ability of the hydrogels. While the control group (blood without hydrogel) exhibited a dark red, homogeneous staining indicating incomplete coagulation and intense erythrocyte lysis, the supernatants of all groups treated with hydrogel (0, 1, and 2 HAMA) transformed into completely transparent and colorless liquids. This visual phenomenon confirms that erythrocyte and platelet populations are securely encapsulated within solid clot matrices fixed to hydrogel surfaces. Quantitatively, the control group maintained a basal BCI value of 100%. Upon contact with pure methacrylated alginate hydrogel (0 HAMA), the BCI value decreased to 7.95%, showing an extremely high statistical significance (***p < 0.001) compared to the control. This rapid initiation of coagulation can be attributed to the inherent properties of the AlgMA backbone, which actively supports platelet aggregation and triggers the intrinsic coagulation pathway through surface-mediated charge interactions. More importantly, covalent hybridization of AlgMA with HAMA further accelerated coagulation kinetics in a dose-dependent manner. The 2 HAMA BCI value was remarkably lower compared to the 1 HAMA BCI value. Statistical analysis confirmed the synergistic role of the HA component by verifying a significant difference (*p < 0.05) between pure AlgMA (0 HAMA) and 2% HAMA loaded networks. This enhanced performance can be attributed to two interconnected mechanisms: (i) Fluid Concentration Effect: As highlighted by the high swelling capacity of these hybrid networks, the highly hydrophilic HAMA segments rapidly absorb the aqueous phase of whole blood upon contact, thereby locally concentrating cellular components (erythrocytes and platelets) and endogenous coagulation factors at the hydrogel interface.^42^ (ii) Biochemical Activation: The presence of HAMA facilitates specific macromolecular interactions with cell surface receptors (such as CD44 expressed in activated platelets), which biochemically stimulates dense platelet aggregation and can stabilize the newly formed fibrin network.^43^ The enhanced hemostatic performance may be attributed to a dual-action mechanism, in which the ionically cross-linked AlgMA network facilitates rapid coagulation through Ca^2+^-mediated activation of the coagulation cascade, while the HAMA component contributes to fluid absorption, structural integrity, and favorable cell–matrix interactions that collectively support clot stabilization and subsequent tissue repair.^44,45^

**Figure 11:**
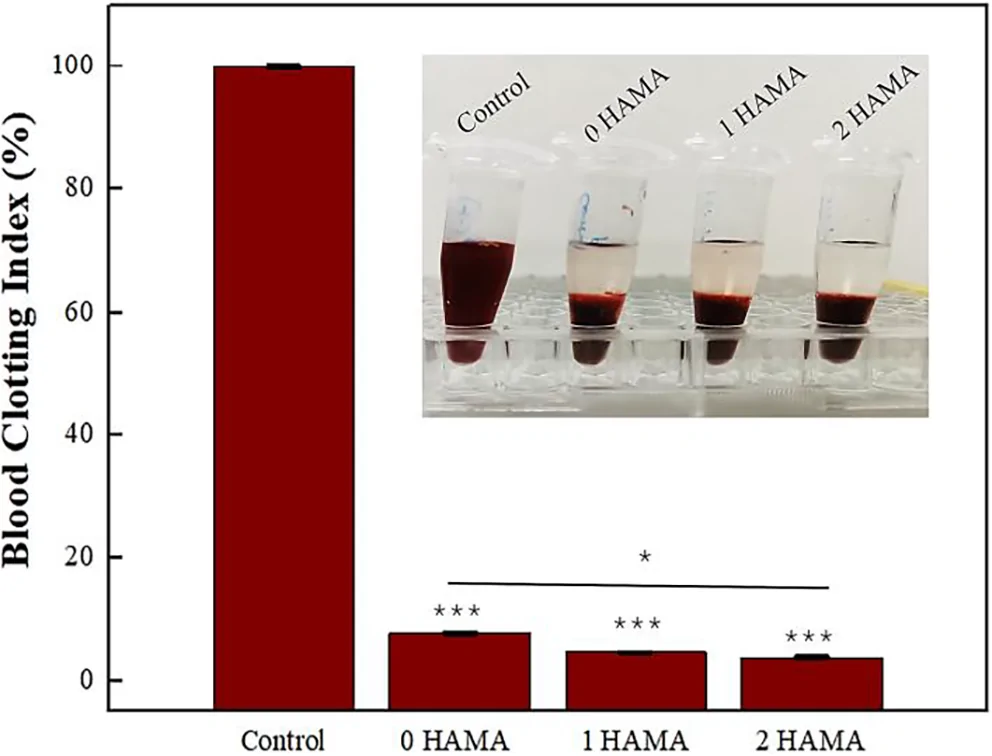
In vitro BCI analysis of AlgMA/HAMA hybrid hydrogels (***p < 0.001 vs Control, and *p < 0.05 between 0 HAMA and 2 HAMA). Inset photograph shows the visual transparency of centrifuged supernatants, demonstrating rapid cell entrapment within the stable clot matrix. Data are expressed as mean ± SD.

## 4 Conclusion

This study focuses on the development of a biocompatible, double cross-linked AlgMA/HAMA hybrid hydrogel designed for traumatic wound healing and acute bleeding control. When exposed to visible light and endogenous calcium ions (Ca^2+^) at the injury site, the system can rapidly gel in situ through cooperative photo-cross-linking and ionic gelation (AlgMA-Ca^2+^) mechanisms. This composite network, confirmed to have high cytocompatibility by MTT and wound scratch tests, shows enormous potential for replacing existing clinical wound dressings. To determine the optimal ratio for clinical applications, a comprehensive comparison was conducted between 0%, 1%, and 2% HAMA formulations. While the 1% HAMA matrix slightly accelerated cellular migration within the first 24 h, the 2% HAMA formulation proved to be the overall best choice for severe traumatic injuries. Traumatic wound management requires matrices that can withstand physiological mechanical loads, resist rapid degradation, and provide an immediate, robust physical barrier against severe bleeding. The 2% HAMA web exceptionally meets these requirements with its high compressive and tensile strength, allowing the matrix to adapt to dynamic tissue movements without structural degradation. Furthermore, controlled swelling kinetics for 24 h and a low degradation rate for 6 weeks establish an ideal balance between mechanical strength and longevity. Most importantly, the clinical safety and active hemostatic mechanism of the optimal matrix have been validated by advanced hematological assessments. The 2% HAMA hydrogel demonstrated high compliance with ISO 10993–4, exhibiting a hemolysis rate of only 1.19%, well below the strictly accepted international safety threshold (<5%). Moreover, the formulation achieved ultrafast coagulation kinetics, remarkably reducing BCI to 3.76%. This quantitative hemostatic performance demonstrated that by combining the active biochemical role of therapeutic Ca^2+^ ion release with synergistic fluid absorption and the platelet aggregation property of the HAMA network, it effectively captures blood components and forms a stable interface clot within seconds. These findings represent a significant contribution to the advancement of individualized regenerative therapies and emergency medicine. While chemical interactions at the hydrogel-tissue interface remain hypothetical at this stage of the study, the system’s highly robust structural performance, biosafety, and rapid hemostatic capability make the 2% HAMA formulation a prime candidate for further in vivo studies of traumatic wound healing and bleeding models.

## Supplementary Material



## Notes

Statistical Analysis for all data (PDF)
