# Supplementary material for: Development of Dual-Cross-Linked AlgMA/HAMA Hybrid Hydrogels for Traumatic Wound Healing
Source: ACS Omega. 2026 Aug 26;11(35):52855–66. doi: 10.1021/acsomega.6c04561 (PMC13564017; doi:10.1021/acsomega.6c04561)
Supplement: Supplementary file 1 [file ao6c04561_si_001.pdf]

Supporting Information for Publication

Development of Dual-Crosslinked AlgMA/HAMA Hybrid Hydrogels for Traumatic Wound Healing

*Simay Y. Rivas Rodriguez[*1,2], Alime Sarıkaya[3,4], Esra Su[5], Cemal Özeroğlu[*1]*

[1] Department of Chemistry, Faculty of Engineering, Istanbul University-Cerrahpasa, Istanbul, 34320, Turkey.

[2] Department of Chemistry, Faculty of Science, Eskisehir Technical University, Eskisehir, 26555, Turkey.

[3] Department of Biotechnology, Institute of Health Science, Bezmialem Vakıf University, 34093, Istanbul, Turkey.

[4] Bezmialem Vakif University Experimental Application and Research Center Cell Culture Laboratory, 34093 Istanbul, Turkey.

[5] Department of Aquatic Biotechnology and Genomics, Faculty of Aquatic Sciences, Istanbul University, 34134 Istanbul, Turkey.

*Corresponding Author(s)

Table S1. Post-Hoc Tukey HSD tests results

Dependent Variable	(A)	(B)	Mean Difference (A-B)	Sig.
Swelling ratio	0 HAMA	1 HAMA	1.200*	0.017
		2 HAMA	-1.920*	0.002
	1 HAMA	0 HAMA	-1.200*	0.017
		2 HAMA	-3.120*	<0.001
	2 HAMA	0 HAMA	1.920*	0.002
		1 HAMA	3.120*	<0.001
Degradation ratio 3w	0 HAMA	1 HAMA	1.915*	<0.001
		2 HAMA	-3.210*	<0.001
	1 HAMA	0 HAMA	-1.915*	<0.001
		2 HAMA	-5.124*	<0.001
	2 HAMA	0 HAMA	3.210*	<0.001
		1 HAMA	5.124*	<0.001
Degradation ratio 6w	0 HAMA	1 HAMA	0.551	0.786
		2 HAMA	3.952*	0.007
	1 HAMA	0 HAMA	-0.551	0.786
		2 HAMA	3.401*	0.014
	2 HAMA	0 HAMA	-3.952*	0.007
		1 HAMA	-3.401*	0.014
Cell viability 24h	0 HAMA	1 HAMA	7.693	0.079
		2 HAMA	6.470	0.136
	1 HAMA	0 HAMA	-7.693	0.079
		2 HAMA	-1.223	0.905
	2 HAMA	0 HAMA	-6.470	0.136
		1 HAMA	1.223	0.905
Cell viability 48h	0 HAMA	1 HAMA	15.970	0.857
		2 HAMA	-1.733	0.998
	1 HAMA	0 HAMA	-15.970	0.857
		2 HAMA	-17.703	0.828
	2 HAMA	0 HAMA	1.733	0.998
		1 HAMA	17.703	0.828
Wound healing 24h	0 HAMA	1 HAMA	-9.904	0.489
		2 HAMA	22.945	0.069
	1 HAMA	0 HAMA	9.904	0.489
		2 HAMA	32.848*	0.016
	2 HAMA	0 HAMA	-22.945	0.069
		1 HAMA	-32.848*	0.016
Wound healing 48h	0 HAMA	1 HAMA	3.052	0.927
		2 HAMA	42.004*	0.005
	1 HAMA	0 HAMA	-3.052	0.927
		2 HAMA	38.952*	0.007
	2 HAMA	0 HAMA	-42.004*	0.005
		1 HAMA	-38.952*	0.007

Tensile E	0 HAMA	1 HAMA	14.057	0.055
		2 HAMA	6.257	0.430
	1 HAMA	0 HAMA	-14.057	0.055
		2 HAMA	-7.800	0.294
	2 HAMA	0 HAMA	-6.257	0.430
		1 HAMA	7.800	0.294
Tensile σ_f	0 HAMA	1 HAMA	-14.303*	0.009
		2 HAMA	-37.390*	<0.001
	1 HAMA	0 HAMA	14.303*	0.009
		2 HAMA	-23.087*	<0.001
	2 HAMA	0 HAMA	37.390*	<0.001
		1 HAMA	23.087*	<0.001
Tensile ε_f	0 HAMA	1 HAMA	-5.563	0.241
		2 HAMA	-47.617*	<0.001
	1 HAMA	0 HAMA	5.563	0.241
		2 HAMA	-42.053*	<0.001
	2 HAMA	0 HAMA	47.617*	<0.001
		1 HAMA	42.053*	<0.001
Compression E	0 HAMA	1 HAMA	-22.427	0.201
		2 HAMA	-146.553*	<0.001
	1 HAMA	0 HAMA	22.427	0.201
		2 HAMA	-124.127*	<0.001
	2 HAMA	0 HAMA	146.553*	<0.001
		1 HAMA	124.127*	<0.001
Compression σ_f	0 HAMA	1 HAMA	39.377	0.156
		2 HAMA	12.937	0.766
	1 HAMA	0 HAMA	-39.377	0.156
		2 HAMA	-26.440	0.375
	2 HAMA	0 HAMA	-12.937	0.766
		1 HAMA	26.440	0.375
Compression ε_f	0 HAMA	1 HAMA	6.800	0.118
		2 HAMA	8.777*	0.049
	1 HAMA	0 HAMA	-6.800	0.118
		2 HAMA	1.977	0.776
	2 HAMA	0 HAMA	-8.777*	0.049
		1 HAMA	-1.977	0.776

* The mean difference is significant at the 0.05 level.
